# NMDA receptor antagonism with novel indolyl, 2-(1,1-Dimethyl-1,3-dihydro-benzo[e]indol-2-ylidene)-malonaldehyde, reduces seizures duration in a rat model of epilepsy

**DOI:** 10.1038/srep45540

**Published:** 2017-03-30

**Authors:** Hussin A. Rothan, Elham Amini, Fadihl L. Faraj, Mojtaba Golpich, Teow Chong Teoh, Khadijeh Gholami, Rohana Yusof

**Affiliations:** 1 Department of Molecular Medicine, Faculty of Medicine, University of Malaya, 50603 Kuala Lumpur, Malaysia; 2 Department of Medicine, Faculty of Medicine, Universiti Kebangsaan, Malaysia Medical Centre (HUKM), 56000 Kuala Lumpur, Malaysia; 3 Department of Chemistry, Faculty of Science, University of Diyala, Diyala Governorate, Iraq; 4 Department of Bioinformatics, Institute of Biological Sciences, Faculty of Science, 50603 Kuala Lumpur, Malaysia; 5 Division of Human Biology, School of Medicine, International Medical University, Kuala Lumpur, Malaysia

## Abstract

N-methyl-D-aspartate receptors (NMDAR) play a central role in epileptogensis and NMDAR antagonists have been shown to have antiepileptic effects in animals and humans. Despite significant progress in the development of antiepileptic therapies over the previous 3 decades, a need still exists for novel therapies. We screened an in-house library of small molecules targeting the NMDA receptor. A novel indolyl compound, 2-(1,1-Dimethyl-1,3-dihydro-benzo[e]indol-2-ylidene)-malonaldehyde, (DDBM) showed the best binding with the NMDA receptor and computational docking data showed that DDBM antagonised the binding sites of the NMDA receptor at lower docking energies compared to other molecules. Using a rat electroconvulsive shock (ECS) model of epilepsy we showed that DDBM decreased seizure duration and improved the histological outcomes. Our data show for the first time that indolyls like DDBM have robust anticonvulsive activity and have the potential to be developed as novel anticonvulsants.

Epilepsy affects around 50 million people worldwide^1^. The last two decades have seen significant advances in new therapies for epilepsy including new antiepileptic drugs (AED)^2^, surgery^3^, cell therapy^4^, gene therapy^5^, and brain stimulation^6^. However, approximately one- third of people with epilepsy continue to have seizures that are intractable and experience intolerable side effects to currently available treatments^7^. Therefore, despite recent therapeutic advances, epilepsy still continues to be a major health problem and more therapies are needed including new anticonvulsant drugs. Although our understanding of epilepetogenesis has improved over the years, the complex array of pathological processes that lead to epilepsy are still largely unknown. Recently, evidence supporting the role of neuroinflammation^8^, oxidative stress and reactive oxygen species (ROS) production^9^, mitochondrial dysfunction^10^, damage of blood–brain barrier (BBB)^11^, as well as failure in the regulation of Gamma-aminobutyric acid (GABA) has been put forth^12^.

It has also been shown that N-methyl-D-aspartate receptors (NMDAR) are important subtype of the glutamate receptors which play a crucial role in both pathological and physiological processes^13^. NMDARs are glutamate-gated cation channel that mediates excitatory neurotransmission in the central nervous system (CNS)^14^. Activation of the NMDARs plays an important role in the pathogenesis of a wide range of neuropathological disorders including schizophrenia, stroke, depression, multiple sclerosis, Huntington’s disease^15^, Parkinson’s disease^16^, Alzheimer’s disease^17^,^18^, and epilepsy^15^,^19^. In epilepsy, NMDA induces seizures in a different pattern compared and causes different long term consequences compared to kainic acid^20^. Signalling activation in neurons results in a rapid increase of NMDA receptor, leading to excitotoxic damage through consequent excessive calcium (Ca2+) influx. Increased Ca2+ fluxes through NMDA channels due to NMDARs phosphorylation appears to be a crucial mechanism underlying the epileptogenesis. Increased intracellular Ca2+ in turn results in pathologic outcomes such as neuronal hyperexcitability or injury, decreased seizure threshold and subsequent necrosis or apoptosis^21^,^22^,^23^. Recent studies demonstrated that the NMDAR is broadly expressed in the cerebral cortex and hippocampus, which is implicated in the pathogenesis of epilepsy^14^,^24^. However, prevention of NMDAR during epileptogenesis reduced epileptic brain injury provoked by status epilepticus in rodent hippocampus^25^. Interestingly, it has been indicated that NMDAR antagonists provide an anticonvulsant activity to suppress seizures in several epilepsy models^26^. In agreement with this, several studies showed that NMDAR antagonists have elicited protective effects against neuronal damage induced by seizures and the subsequent development of epilepsy^27^,^28^.

Herein, An *in silico* screening was performed using in-house library of five Quinazoline derivatives and five Indolyl derivatives targeting the NMDA receptor. A novel indolyl, [2-(1,1-Dimethyl-1,3-dihydro-benzo[e]indol-2-ylidene)-malonaldehyde], (DDBM) showed the best binding with the NMDA receptor and the computational docking data showed that DDBM antagonised the binding sites of the NMDA receptor at lower docking energies compared to other molecules. Using a rat electroconvulsive shock (ECS) model of epilepsy, The DDBM compound showed considerable decrease in the seizure duration and improvement in the histological outcomes.

## Results

### Generation of DDBM

**Figure 1:**
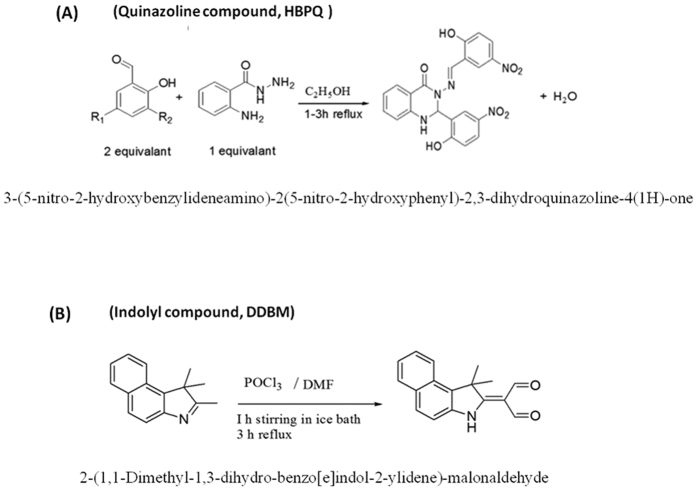
Chemical structures of the selected compounds. (**A**) Chemical synthesis of Quinazoline compoud, HBPQ and the structural data were previously reported 18, 31. (**B**) The Indole compound, DDBM has been synthesized by Vilsmier Haack reaction method. The compound was produced in a good yield via a reaction of 1,1,2-Trimethyl-1H-benzo[e]indole, dimethylformamide and phosphorus trichloride.

We previously reported the chemical synthesis and the structural data of Quinazoline molecules^29^ as presented in Fig. 1A. The DDBM, an Indolyl molecule, was synthesized by the Vilsmier Haack reaction and produced via a reaction of 1,1,2-Trimethyl-1H-benzo[e]indole, dimethylformamide and phosphorus trichloride (Fig. 1B).

### Structural data of DDBM

**Figure 2:**
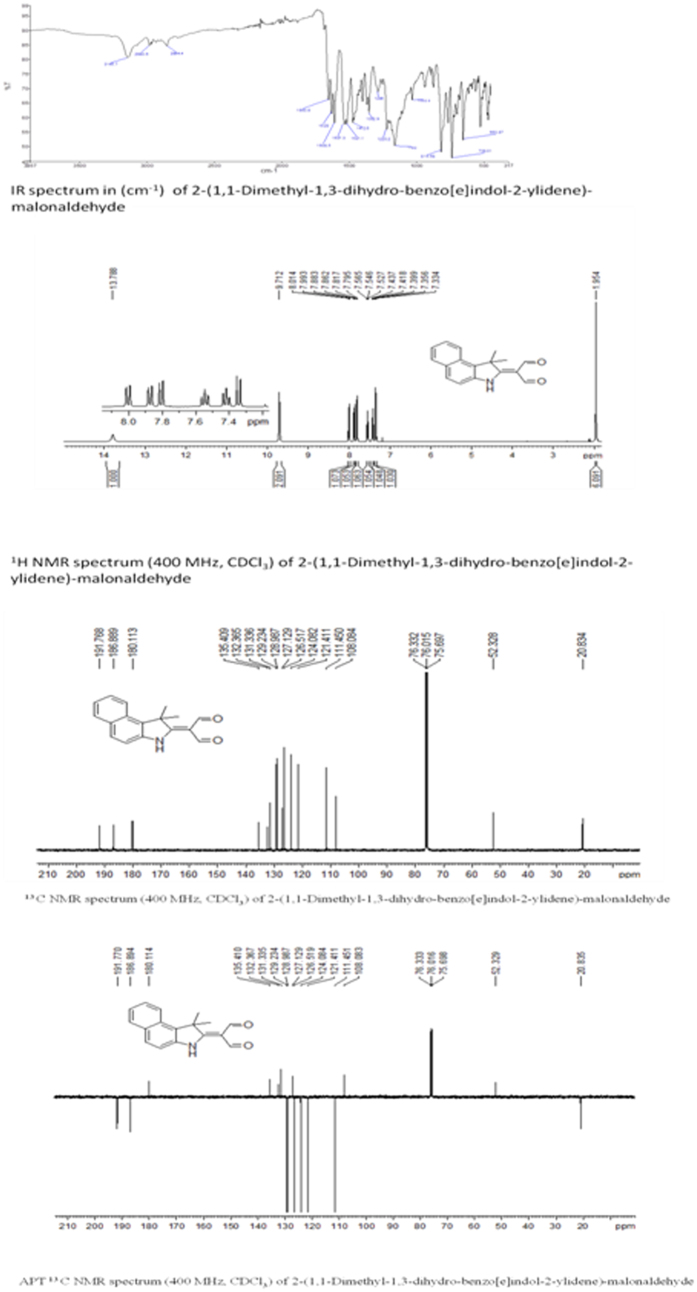
IR Spectral Study of 2-(1,1-Dimethyl-1,3-dihydro-benzo[e] indol-2-ylidene)-malonaldehyde (DDBM). The results showed the main absorption bands (stretching and bending vibrations) such as NH absorption band at 3145 cm^−1^ and carbonyl groups at1650 cm^−1^ and 1628. In addition, C=C group at 1608 cm^−1^. All these main absorption bands were in a good agreement with the proposed structure to confirm the formation of 2-(1,1-Dimethyl-1,3-dihydro-benzo[e]indol-2-ylidene)-malonaldehyde.

The NMR data (1H-NMR and 13C-NMR spectra) of DDBM were recorded in deuterated CDCl3 with chemical shifts expressed in ppm using tetramethylsilane TMS as internal standard. 1H-NMR spectra displayed signal at 13.78 ppm was assigned to (N-H) group of indole ring. A singlet signal appeared at 9.71 ppm was attributed to two protons of C=O carbonyl group. Signals were appeared in the region between 8.01 to 7.33 ppm which assigned to six protons of aromatic rings. As well as a signal at 1.95 ppm attributed to the 6 proton atoms of the two methyl groups. In addition, 13 C NMR results confirmed the 1H-NMR spectral results. Two signals appeared at 191.76 and 186.88 ppm which attributed to C=O carbonyl groups. Ten signals were observed in region between 135.40 to 111.45 ppm belong to carbon atoms of aromatic rings. In addition, three signals at 108.08, 52.32 and 20.82 ppm which attributed to aliphatic carbon atoms (Fig. 2).

Finally APT 13 C NMR result was Supported 1H-NMR and 13C-NMR results and which showed a number of carbon atoms bearing protons of CH2 groups and the quartenary carbon atoms including the solvent carbon appeared at the positive side (above base line), and a number of carbon atoms bearing protons of CH and CH3 appeared at the negative side (under base line). All NMR results were in good agreement to number of hydrogen and carbon atoms with proposed structure of 2-(1,1-Dimethyl-1,3-dihydro-benzo[e]indol-2-ylidene)-malonaldehyde.

### Molecular Docking of test compounds to NMDA receptor

Using an in-house small molecule library consisted of 5 Quinazoline and 5 Indolyl new compounds, we screened these molecules for affinity to bind to the NMDAR. Molecular docking studies showed that Quinazoline compound, 3-(5-nitro-2-hydroxybenzylideneamino)-2(5-nitro-2-hydroxyphenyl)-2,3-dihydroquinazoline-4(1H)-one, (HBPQ) and an indolyl compound, 2-(1,1-Dimethyl-1,3-dihydro-benzo[e]indol-2-ylidene)-malonaldehyde, (DDBM) were the best candidates (Table 1). The DDBM compound exhibited lower docking energy compared with the standard agonist, 1-aminocyclopropane-1-carboxylic acid (ACPC) in binding with chain A as shown in Table 1, but shows approximately half the inhibition compared to agonist trans-1-aminocyclobutane-1,3-dicarboxylic acid (t-ACBD) on chain D. Thus, chain A is a better target for DDBM and at lower extent chain D. This feature for DDBM is important to avoid the possible side effects that have been reported due to complete inhibition of chain D. Thus, we selected DDBM for further investigations because it showed better binding to chain A (Glycine binding site) and less binding to chain D (Glutamate binding site) compared to HBPQ.

Further docking studies was conducted to test whether the DDBM compound can bind with other receptors such as Gamma-aminobutyric acid receptor (GABAR) and α-amino-3-hydroxy-5-methyl-4-isoxazolepropionate receptor (AMPAR). The results showed considerable binding affinity of DDBM with GABAR (−21.0 ± 1.3 kcal/mol) compared to Benzamidine as a standard agonist (−1.6 ± 1.5 kcal/mol). Whereas, the binding affinity of DDBM to AMPAR (−18.4 ± 4.7 kcal/mol) was lower than the standard Perampanel (−29 ± 8.5 kcal/mol) as presented in Table 2.

**Figure 3:**
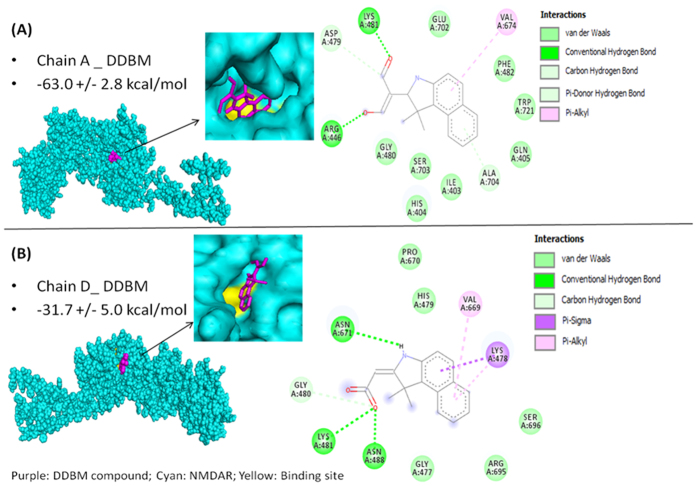
Molecular Docking of DDBM to NMDA receptor. (**A**) Molecular surface rendering of DDBM compound binding to NMDA chain A receptor binding cleft. Van der Waals (vdw) rendering of DDBM binding to NMDA chain A receptor binding cleft. Cyan: NMDA receptor; magenta: ligand; yellow: chain A binding site aa residues. The 2-D diagram of DDBM binding to NMDA chain A showing 1 vdw, 2 hydrogen bonds and 1 pi-alkyl interactions. Docked energy, −63.0 ± 2.8 kcal/mol. (**B**) Molecular surface rendering of DDBM binding to NMDA chain D receptor binding cleft. Van der Waals rendering of DDBM binding to NMDA chain D receptor binding cleft. Cyan: NMDA receptor; magenta: ligand; yellow: chain D binding site aa residues. The 2-D diagram of DDBM binding to NMDA chain D showing 1 vdw, 3 hydrogen bonds and 3 pi-alkyl interactions. Docked energy = −31.7 ± 5.0 kcal/mol.

Figure 3 shows the molecular surface and van der Waals (vdw) molecular representations and 2-D diagram interactions of DDBM to the NMDA receptor chain A and chain D binding sites, respectively. The results showed that the binding of DDBM formed a more well distributed hydrogen bonds on the two separate O atoms on DDBM, hence the ligand-receptor complex formed by chain A is more stable. The more negative docked energy is due to the chain A buried surface is 562.0 ± 6.9 Å2, while chain D is 497.0 ± 95.7 Å2, higher buried surface contributes to more negative desolvation energy. Larger negative desolvation energy increases the interactions and entropy of ligand and receptor, hence making the ligand-receptor complex more thermodynamically favourable. Among all the test compounds, DDBM compound served as the most potential inhibitor for chain A and showed partial inhibition towards chain D.

### Anticonvulsant effects of DDBM compound

**Figure 4:**
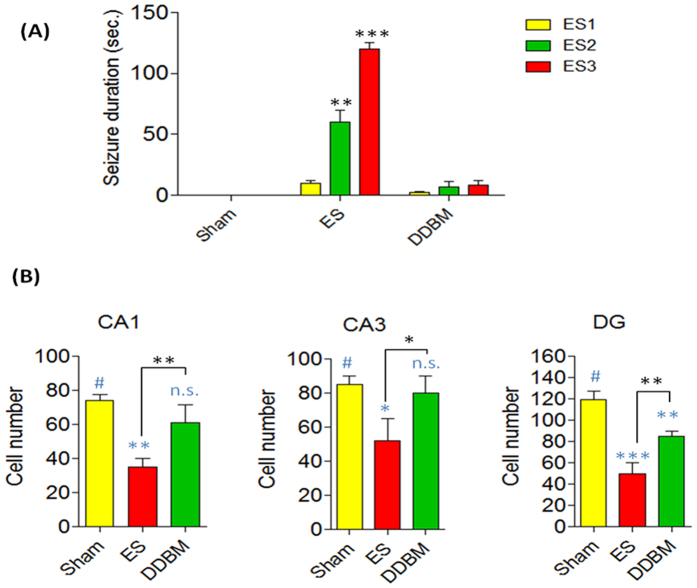
Behavioural and histopathological changes after treatment with DDBM. (**A**) The animals exposed to ECS administration showed varied durations of seizures (6.3 ± 1.8, 69.0 ± 18.1 and 122 ± 7.5 sec for ECS1, ECS2 and ECS3 respectively). Treatment with DDBM showed significant reduction in the duration of seizures of ECS2 (7.1 ± 1.3 sec; p < 0.01) and ECS3 (8.7 ± 1.5 sec; p < 0.001) while the significant effect was not seen in the duration of seizures of ECS1 (2.1 ± 0.3; p > 0.05). The data of this experiment showed clearly that the treatment with DDBM attenuated the ECS-induced seizure durations particularly those induced by ECS2 & ECS3 (Two-way ANOVA with Bonferroni post-tests). (**B**) The number of neurons present in the various hippocampal regions of DDBM- treated and non-treated epileptic rats. In the rats treated with the compound followed by ECS, there were significant differences in the number of neurons present in the CA1 (p < 0.01), CA3 (p < 0.05), and DG (p < 0.01) hippocampal fields, compared to the ECS group. Moreover, significant differences have been observed in this case between the hippocampal regions in the shame and ECS group (CA1, p < 0.01; CA3, p < 0.05; and DG, p < 0.001, One-Way ANOVA with Tukey post test).

The anticonvulsant effects of DDBM against seizures elicited by electroconvulsive shock (ECS) were measured. The animals treated with DDBM performed significantly better on the behavioural symptoms of seizure (e.g. duration) compared with controlled rats. DDBM was administered 6 hours before the first ECS. This type of behaviour built-up progressively into motor limbic seizures that recurred repeatedly and rapidly developed into status epilepticus (SE). While the animals in sham group showed no signs of epilepsy, the ECS-administrated groups underwent varied durations of seizures. The ECS-administrated groups without treatment with DDBM showed durations of seizures 6.3 ± 1.8, 69.0 ± 18.1 and 122 ± 7.5 sec for ECS1, ECS2 and ECS3 respectively. Interestingly, the duration of seizures in the animals treated with DDBM compound were significantly reduced (2.1 ± 0.3, 7.1 ± 1.3 and 8.7 ± 1.5 sec for ECS1, ECS2 and ECS3 respectively). The data of this experiment showed clearly that the treatment with DDBM compound attenuated the ECS-induced seizure durations particularly those induced by ECS2 & ECS3 (Fig. 4A).

### Morphological and neurodegenerative changes in the hippocampus

The morphological changes were especially visible in the CA1 & DG hippocampal sectors compare with CA3 hippocampal sector of the ECS model of epilepsy. A qualitative analysis of brain histological sections in the DDBM- treated rats showed markedly decreased intensity of neurodegenerative changes in the CA1, CA3 and DG hippocampal fields (Fig. 4B).

A quantitative analysis of histological sections showed a marked neuroprotective effect of DDBM. In the rats treated with DDBM followed by ECS, there were significant differences in the number of neurons present in the CA1 (p < 0.01), CA3 (p < 0.05), and DG (p < 0.01) hippocampal fields, compared to the control ECS group. Moreover, significant differences have been observed in this case between the hippocampal regions in the sham and ECS group (CA1, p < 0.01; CA3, p < 0.05; and DG, p < 0.001, One-Way ANOVA with Tukey post test). The number of neurons present in the CA1 hippocampal field in the sham, ECS-induced epileptic rats and treatment groups were 74 ± 2.1, 35.5 ± 6.3, 61 ± 5.2 neurons, respectively, whereas in the CA3 hippocampal field they were 85 ± 2.8, 52 ± 7.5, 80.0 ± 5.7 neurons, respectively. Meanwhile, they reached 119.0 ± 4.9, 50.0 ± 5.8, 87 ± 1.4, and 85.0 ± 2.9 neurons, respectively in the DG hippocampal field (Fig. 4B).

## Discussion

**Figure 5:**
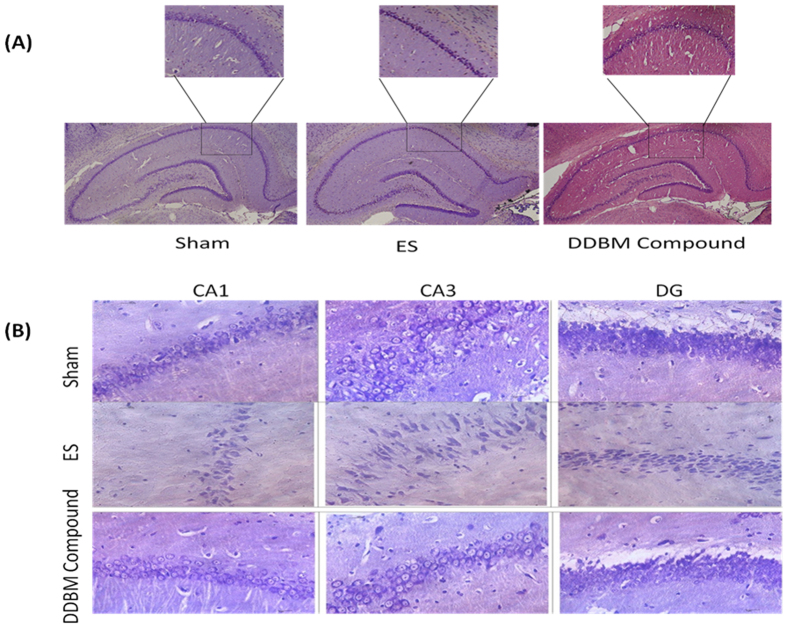
Morphological change in hippocampus region after treatment of epileptic rats with DDBM. Original magnification, x100 (**A**). Neurodegenerative changes in field CA1, CA3, & DG of the hippocampus in the DDBM- treated and non-treated rats in the ECS model of epilepsy. Original magnification x400 (**B**).

Seizure control can have a profound effect on the quality of life of people with epilepsy and also reduces morbidity and mortality. It has also been shown that prolonged seizures may play an important role in hippocampal neuronal loss, which may intern potentiate epileptogenesis^30^. Several animal models of epilepsy are used in epilepsy research including the development of new antiepileptic drugs^31^. The ECS is a well-established model that induces generalized tonic-clonic seizure activity^32^ and rapidly triggers inflammatory responses in glial cells in brain regions affected by epileptic activity^33^. Furthermore, The ECS model that was used in this study also allows for measurement of the neuroprotective effect of a particular drug, independent of any anticonvulsant activity. The markers of injury can be discerned by measurement of the degree of morphological changes and the extent of neuronal loss^34^ as explained by Figs 4 and 5 in our study. In addition, seizure induces cell death and other events that induce neurodegeneration result from over-activation of ionotropic glutamate receptors which leads to increased intracellular levels of Ca+2 and Na+ and causes swelling and cell lysis. There is also energy failure, production of free radicals, activation of enzymatic complex, and cell death (both necrosis and apoptosis)^35^.

In this study, we screened an in-house library of small molecules to target NMDAR, a heteromeric ion channel that initiates seizures in human epilepsy^36^. The DDBM molecule, an indole derivative, that showed the best binding with NMDA receptor was characterised by IR spectra34, 1H and 13C NMR spectra35. The computational docking data showed that DDBM interfered with the binding sites of NMDA receptor at lower docking energy compared to other compounds. This compound was able to bind with NMDA subunits (NR1 and NR2) binding clefts via hydrogen bonds, Van der Waals and pi-alkyl interactions leading to interfere with original ligands, glycine and glutamate. The results of this study showed that the binding of DDBM with NR1 (−63 kcal/mol) and NR2 (−31 kcal/mol) might eventually lead to reduced NMDAR activity by impairing the binding of glycine and glutamate to the NR1 and NR2 subunits, respectively^37^.

Many published *in vivo* studies in epilepsy have reported that NMDAR antagonists have potent anticonvulsant actions^38^. We assumed that the oral administration of the test compound prior to exposure of the animals to ECS would interfere with the NMDAR and inhibit the initiation of seizures. Treated animals that were exposed to three courses of ECS showed significant reduction in epileptic seizures compared to the ECS control group. Administration of DDBM at six hours prior to ECS induction showed the highest reduction in epileptic seizures. This time interval may be necessary for optimal absorption of the test compound and inhibit the induction of epileptic seizures. Quantitative analysis of hippocampal histological sections confirmed a considerable neuroprotective effect of DDBM.

Evidence that single or repeated brief seizures could cause neuronal death emerged from work in animals using electrical stimulation of various brain regions. It has been showed repeated stimulation of the perforant path, olfactory bulb or amygdala resulted in progressive decreases in neuronal density in multiple subfields of the hippocampus, including the hilus, CA1 and CA3, and parts of the entorhinal cortex^30^. These findings confirm that brief evoked seizures in different levels and properties can cause different level of neuronal death in various strains of animal models. Indeed, repeated exposures to ECS using high currents induces a kindling effect in that the tonic-clonic convulsion response becomes increasingly severe^39^. Consistent with these findings, in our study, cell morphology and numbers of CA1, CA3 and DG of animals treated with DDBM were significantly enhanced compared to untreated animals after ECS administration. The seizure-induced neuronal damage and apoptosis normally results from excitotoxic, glutamatergic neurotransmission, excessive Na+ and Ca2+ ions^40^. Thus reducing the activity of NMDA receptor by administrating DDBM may lead reduction in the excitotoxic effect of NMDAR and the osmolytic stress and cellular free-radical production, culminating in necrosis of neurones^40^.

Implement of a convenient technique that can show the actual binding affinity of DDBM compound with NMDAR can be considered as the main limitation of this study. We proposed this binding based on our *in silico* study and the considerable activity of the DDBM compound as anticonvulsant agent. Therefore, further investigation is required to illustrate the binding affinity of DDBM compound to NMDAR and the possible interaction with other receptors involved in the development of epilepsy.

In conclusion, we identified a novel antiepileptic (anticonvulsant) molecule and described its chemical synthesis and ability to interfere with both binding sites of NMDAR. This molecule could be a potent lead for more investigations towards finding novel therapeutics against epilepsy and other neurodiseases related to excitotoxic effects of NMDAR.

## Methods

### Synthesis of DDBM compound, 2-(1,1-Dimethyl-1,3-dihydro-benzo[e]indol-2-ylidene)-malonaldehyde

All the solvents and reagents in this research were purchased from Merck, Sigma-Aldrich and fisher scientific. Melting points of the synthesized compound was determined by open capillary tubes melting point apparatus and are uncorrected used without further purification and are uncorrected. The purity of the compounds was checked using pre-coated TLC plates MERCK, 60F254 using hexane: ethyl acetate (4:1) as an eluent. The developed chromatography plates were visualized under UV-Vis at 254 nm., 1H and 13C-NMR spectra were recorded on AVN Bruker 400 FT–NMR system. Tetramethylsilane TMS was used as an internal standard, deuterated CDCl3 was used as a solvenst for NMR spectrophotometer. Elemental analysis CHN was performed on an elemental analyzer Perkin Elmer CHNS/O 2400 series II.

A solution of 1,1,2-Trimethyl-1H-benzo[e]indole (5 g, 23.9 mmol), in 15 ml anhydrous dimethylformamide was cooled in an ice bath. A solution of (8.78 ml, 95.6 mmol) phosphoryl chloride in anhydrous dimethylformamide 15 ml was cooled in an ice bath was added to the first solution drop wise with stirring over a period of 1 h at below 5 °C. The cooling bath was removed and the reaction mixture was stirred at 85 °C for 3 h. The resulting mixture was poured on to ice water, the pH was adjusted to 8.0 by the addition of aqueous NaOH (35%) whereupon the solid product was precipitated. It was filtered, washed with hot water, dried in oven to afford solid product. The solid product recrystallized from ethanol to give pale yellow crystals of 2-(1,1-Dimethyl-1,3-dihydro-benzo[e]indol-2-ylidene)-malonaldehyde.

Yield: (5.8 g, 91%) m.p 199–200. Anal. Calc. for C_17_H_15_NO_2_ (265.31): C, 76.96; H, 5.70; N, 5.28. Found: C, 76. 65; H, 5.59; N, 5.38. IR data (cm^−1^): 3145 ʋ(N-H), 2980 ʋ(C-H aromatic), 2854 ʋ(C-H aliphatic) 1650 and 1628 ʋ(C=O), 1608 ʋ(C=C), 1220 ʋ(C-N) and 738 ʋ(C-H bending). ^1^H NMR (400 MHz, CDCl_3_, ppm): *δ* = 13.78 (s, 1H, N*H*), 9.71 (s, 2H, C*H*O), 8.00 (d, 1H, Ar-*H*), 7.87 (d, H, Ar-*H*), 7.80 (d, 1H, Ar-*H*) 7.54 (t, 1H, Ar-*H*), 7.41 (t, 1H, Ar-*H*), 7.34 (d, 1H, Ar-*H*), 1.95 (s, 6H, 2x C*H*_3_) and 1.58(s, 2H, 2xC*H*). ^13^C NMR (400 MHz, CDCl_3_, ppm): *δ* 191.76 and 186.89 ppm (2x*C*HO), 180.11 ppm (N-*C*=C), 135.40 ppm, 132.36 ppm, 131.33 ppm, 129.23 ppm, 128.98 ppm, 127.12 ppm, 126.51 ppm, 124.08 ppm, 121.41 ppm and 111.45 ppm (*Ar*-H), 108.06 ppm (C=*C*-C=O), 52.32 ppm (CH_3_-*C*-CH_3_) and 20.83(2x *C*H_3_).

### Molecular modelling of ligands

NMDAR, GABAR and AMPAR data were obtained from Protein Databank (PDB: 4TLL, 4COF and 5L1F respectively). The two binding sites of NMDAR [1-aminocyclopropane-1-carboxylic acid (ACPC) binding site on chain A and trans-1-aminocyclobutane-1,3-dicarboxylic acid (t-ACBD) binding site on chain D]^41^, the binding sites of GABAR [Benzamidine]^42^ and AMPAR [Perampanel]^43^ were indentified.

The ligands HBPQ and DDBM were modelled by Discovery Studio Client v4.5.0.15071 and minimized by CHARMm force field (Accelrys Inc., Dassault Systèmes, BIOVIA Corp., San Diego, CA, USA) were identified.

### Molecular docking of NMDA receptor

The minimized ligands were targeted individually on two binding sites of NMDA receptor at F482, T516, W721, D722 for chain A and H479, R512, Y745 for chain D respectively^42^. Haddock 2.2 molecular docking software web server was used to perform the docking simulation using the protein-ligand module^44^,^45^. The lowest docked energy ± standard deviation was extracted. The docked conformation graphics was generated using PyMOL 1.3 ((TM) Educational Product – Copyright © 2010 Schrodinger, LLC) and the 2-D diagram was computed by using Discovery Studio Client v4.5.0.15071 for the van der Waals, hydrogen bonding and pi interactions.

### Animals

Experiments were carried out on male Sprague-Dawley rats weighing 200–250 g obtained from the Animal Experimental Unit - University of Malaya (UM) and housed in the satellite animal holding laboratory, at the Departments of Pharmacology - UM. Animals were kept under a 12 hrs-12 hrs light/dark cycle and allowed free access to food and water. All experiments were performed in a silent room at 22–24 °C. All procedures were carried out with the approval from the Institutional Animal Care and Use Committee (IACUC), University of Malaya and all the experiments in this study were performed in accordance with relevant guidelines and regulations. The animals were randomly divided into experimental groups, (a) sham group, a control group which were intact with no previous experimental history; (b) ECS group, which only received a chronic ECS administration; (c) treated groups, which were given the compound at 6 hours before administration of ECS. All efforts were made to minimize animal suffering and the number of animals required (n = 5 per group).

### Animal model for epilepsy

The current model of epilepsy is similar to previous studies^30^,^46^ with minor modifications. The minimal current, duration, frequency, and interval required to produce a tonic-clonic seizure in the rat is strain-dependent^47^,^48^. To determine a threshold current, duration, frequency, and interval for seizure in the male SD rat, we applied a preliminary study. In brief, rats received only 1 shock with the same current and duration used for previous studies (60 mA, 50 Hz, 1 sec). Since we have not observed the tonic-clonic seizure, we exposed them to the second shock with the same current and duration after three hours. Due to no response observed from some of the animals, we repeated it again after another three hours. The third course of ECS induced the tonic-clonic seizures in all animals. Therefore, the current protocol designed based on our dose-finding experiments. In current study, we aimed to induce tonic-clonic seizure using three repeated seizures (60 mA, 50 Hz, 1 sec). Interval between the ECS seizures was set only 3 hrs apart; a period during which the cell loss were observed in the hippocampus. This time window was selected based on the time course of the increase in proinflammatory cytokines which have been previously reported^49^.

### *In vivo* study

In this study, we considered that the ECS administration caused severe pain to animals. Therefore, to reduce animal numbers, first, we conducted acute toxicity experiment and a pilot experiment to optimize the effective dose of the test compound and the period of drug treatment before ECS administration. Animals that did not display tonic-clonic seizure in response to a course of three ECS were excluded from experiments as non-responders. Thus, all animals allocated for the non-treated and treated groups (all groups except sham) showed the tonic-clonic seizure(s). However, the seizure duration, intensity, and frequency were different among them.

Then, a single dose was used to determine the potential effect of the test compound. All rats in treated groups were given 1 ml (100 mg/Kg) of DDBM or 1 ml normal saline via oral gavage, 6 hrs before first administration of the first ECS. Sham treated animals received 1 ml normal saline. Seizures were induced by administration of the chronic ECS. The first ECS (60 mA, 50 Hz, 1 sec duration) was applied via bilateral ear clip electrodes using an ECS apparatus (Electroconvulsive Shock Unit, USA) to evoke a tonic seizure, followed by clonic seizures 38. Animals received a course of three ECS (ECS1, ECS2, & ECS3) seizures, administered at 3 hrs intervals. The animals were observed for 1 hour after induction of the last ECS (ECS3). An assessment of ECS-induced convulsions was performed by measuring the duration of seizures from the shock initiation to the last convulsive movements in each group. The sham group were applied the ear clip electrodes but without any current passing.

### Tissue Preparation and Histology

Twenty four hours after the last ECS or sham session, the rats were anesthetized with CO2 and then decapitated using a guillotine. The brains were quickly removed and placed on ice. Brain samples were fixed with a fixative solution containing 4% freshly prepared paraformaldehyde (Sigma, USA) in pH 7.4, at least for 10 days. Brain tissue) wasparaffin embedding (Leica, Germany). 5 μm sections were cut (Leica, Germany). After being dewaxed and rehydrated, the brain tissue sections were immersed in 0.5% cresyl violet (Sigma, USA), rinsed with distilled water, dehydrated in ethanol solutions with increasing concentrations, and cleared in xylene. Next, the sections were mounted with Permount coverslip and observed under a light microscope (Nikon). Photographs of the histological preparations were taken in magnification x400. The cells were counted with the ImageJ 1.50b software using the Cell Counter function. This function allows manual cell counting and simultaneous marking of the already counted cells, which prevents calculation mistakes. Ten calculations from each of the three randomly selected fields from hippocampal sectors CA1, CA3 and dentate gyrus (DG) from either brain hemisphere were analyzed in all the animals.

### Statistical Analysis

Values are expressed as means ± SEM of five experiments, with all measurements performed in duplicate. Analyses of variance (Two- Way ANOVA and One- Way ANOVA) were performed to compare results between different groups. The Bonferroni and Tukey post test were performed as a post hoc test, the level of significance is p < 0.05.

## Additional Information

**How to cite this article:** Rothan, H. A. *et al*. NMDA receptor antagonism with novel indolyl, 2-(1,1-Dimethyl-1,3-dihydro-benzo[e]indol-2-ylidene)-malonaldehyde, reduces seizures duration in a rat model of epilepsy. *Sci. Rep.* **7**, 45540; doi: 10.1038/srep45540 (2017).

**Publisher's note:** Springer Nature remains neutral with regard to jurisdictional claims in published maps and institutional affiliations.

## Figures and Tables

**Table 1:** Docking energy of test compounds to chain A and chain D of NMDA receptor, with the agonist compounds as reference.

Compounds	Chain A Energy (kcal/mol)	Chain D Energy (kcal/mol)
DDBM	−63. 0 ± 2.8	−31.7 ± 5.0
HBPQ	−56.6 ± 4.6	−41.0 ± 6.3
*ACPC	−65.2 ± 3.7	—
**t-ACBD	—	−53.6 ± 2.8

^*^Reference compound binds to chain A, 1-aminocyclopropane-1-carboxylic.

^**^Reference compound binds to chain D, trans-1-aminocyclobutane-1,3-dicarboxylic acid.

**Table 2:** Docking energy of DDBM compound to NMDA receptor (chain A and chain D), GABA receptor and AMPA receptor.

Receptors	Binding energy of DDBM	Binding energy of the reference
	(kcal/mol)	(kcal/mol)
NMDA	−63.0 ± 2.8	−65.2 ± 3.7
Chain A		1-aminocyclopropane-1-carboxylic acid
NMDA	−31.7 ± 5.0	−53.6 ± 2.8
Chain D		trans-1-aminocyclobutane-1,3-dicarboxylic acid
GABA	−21.0 ± 1.3	−1.6 ± 1.5
		Benzamidine
AMPA	−18.4 ± 4.7	−29.8 ± 8.5
		Perampanel

## References

[b1] Fabene P. F., Bramanti P. & Constantin G. The emerging role for chemokines in epilepsy. J Neuroimmunol. 224, 22–27 (2010). 20542576 10.1016/j.jneuroim.2010.05.016

[b2] Hwang H. & Kim K. J. New antiepileptic drugs in pediatric epilepsy. Brain Dev. 30, 549–555 (2008). 18328657 10.1016/j.braindev.2008.01.007

[b3] Asano E., Juhasz C., Shah A., Sood S. & Chugani H. T. Role of subdural electrocorticography in prediction of long-term seizure outcome in epilepsy surgery. Brain. 132, 1038–1047 (2009). 19286694 10.1093/brain/awp025 PMC2668945

[b4] Sebe J. Y. & Baraban S. C. The promise of an interneuron-based cell therapy for epilepsy. Dev Neurobiol. 71, 107–117 (2011). 21154914 10.1002/dneu.20813 PMC3059084

[b5] Naegele J. R., Maisano X., Yang J., Royston S. & Ribeiro E. Recent advancements in stem cell and gene therapies for neurological disorders and intractable epilepsy. Neuropharmacology. 58, 855–864 (2010). 20146928 10.1016/j.neuropharm.2010.01.019 PMC2838966

[b6] Fridley J., Thomas J. G., Navarro J. C. & Yoshor D. Brain stimulation for the treatment of epilepsy. Neurosurg Focus. 32, E13 (2012). 10.3171/2012.1.FOCUS11334 22380854

[b7] Zeng L. H., Rensing N. R. & Wong M. The mammalian target of rapamycin signaling pathway mediates epileptogenesis in a model of temporal lobe epilepsy. J Neurosci. 29, 6964–6972 (2009). 19474323 10.1523/JNEUROSCI.0066-09.2009 PMC2727061

[b8] Somera-Molina K. C., Nair S., Van Eldik L. J., Watterson D. M. & Wainwright M. S. Enhanced microglial activation and proinflammatory cytokine upregulation are linked to increased susceptibility to seizures and neurologic injury in a ‘two-hit’ seizure model. Brain Res. 1282, 162–172 (2009). 19501063 10.1016/j.brainres.2009.05.073 PMC2739829

[b9] Shin E. J. . Role of oxidative stress in epileptic seizures. Neurochem Int. 59, 122–137 (2011). 21672578 10.1016/j.neuint.2011.03.025 PMC3606551

[b10] Folbergrova J., Jesina P., Haugvicova R., Lisy V. & Houstek J. Sustained deficiency of mitochondrial complex I activity during long periods of survival after seizures induced in immature rats by homocysteic acid. Neurochem Int. 56, 394–403 (2010). 19931336 10.1016/j.neuint.2009.11.011

[b11] Van Vliet E. A. . Blood-brain barrier leakage may lead to progression of temporal lobe epilepsy. Brain. 130, 521–534 (2007). 17124188 10.1093/brain/awl318

[b12] Huberfeld G. . Perturbed chloride homeostasis and GABAergic signaling in human temporal lobe epilepsy. J Neurosci. 27, 9866–9873 (2007). 17855601 10.1523/JNEUROSCI.2761-07.2007 PMC6672644

[b13] Moddel G. . The NMDA receptor NR2B subunit contributes to epileptogenesis in human cortical dysplasia. Brain research 1046, 10–23, doi: 10.1016/j.brainres.2005.03.042 (2005). 15890316

[b14] Zhu X. . NMDA receptor NR2B subunits contribute to PTZ-kindling-induced hippocampal astrocytosis and oxidative stress. Brain research bulletin 114, 70–78, doi: 10.1016/j.brainresbull.2015.04.002 (2015). 25896886

[b15] Hansen K. B. . Implementation of a fluorescence-based screening assay identifies histamine H3 receptor antagonists clobenpropit and iodophenpropit as subunit-selective N-methyl-D-aspartate receptor antagonists. The Journal of pharmacology and experimental therapeutics 333, 650–662, doi: 10.1124/jpet.110.166256 (2010). 20197375 PMC2879924

[b16] Aarsland D. . Memantine in patients with Parkinson’s disease dementia or dementia with Lewy bodies: a double-blind, placebo-controlled, multicentre trial. The Lancet. Neurology 8, 613–618, doi: 10.1016/S1474-4422(09)70146-2 (2009). 19520613

[b17] Tariot P. N. Contemporary issues in the treatment of Alzheimer’s disease: tangible benefits of current therapies. The Journal of clinical psychiatry 67 Suppl 3, 15–22 quiz 23 (2006). 16649847

[b18] Winblad B., Jones R. W., Wirth Y., Stoffler A. & Mobius H. J. Memantine in moderate to severe Alzheimer’s disease: a meta-analysis of randomised clinical trials. Dementia and geriatric cognitive disorders 24, 20–27, doi: 10.1159/000102568 (2007). 17496417

[b19] Chen Q. . Differential roles of NR2A- and NR2B-containing NMDA receptors in activity-dependent brain-derived neurotrophic factor gene regulation and limbic epileptogenesis. J Neurosci. 27, 542–552 (2007). 17234586 10.1523/JNEUROSCI.3607-06.2007 PMC6672795

[b20] Stafstrom C. E. & Sasaki-Adams D. M. NMDA-induced seizures in developing rats cause long-term learning impairment and increased seizure susceptibility. Epilepsy Res. 53, 129–137 (2003). 12576174 10.1016/s0920-1211(02)00258-9

[b21] Lee S. T. . Memantine reduces hematoma expansion in experimental intracerebral hemorrhage, resulting in functional improvement. Journal of cerebral blood flow and metabolism: official journal of the International Society of Cerebral Blood Flow and Metabolism 26, 536–544, doi: 10.1038/sj.jcbfm.9600213 (2006). 16107786

[b22] Picconi B. . NR2B subunit exerts a critical role in postischemic synaptic plasticity. Stroke 37, 1895–1901, doi: 10.1161/01.STR.0000226981.57777.b0 (2006). 16741178

[b23] Vezzani A., Friedman A. & Dingledine R. J. The role of inflammation in epileptogenesis. Neuropharmacology 69, 16–24, doi: 10.1016/j.neuropharm.2012.04.004 (2013). 22521336 PMC3447120

[b24] Di Maio R., Mastroberardino P. G., Hu X., Montero L. & Greenamyre J. T. Pilocapine alters NMDA receptor expression and function in hippocampal neurons: NADPH oxidase and ERK1/2 mechanisms. Neurobiology of disease 42, 482–495, doi: 10.1016/j.nbd.2011.02.012 (2011). 21397025

[b25] Frasca A. . Misplaced NMDA receptors in epileptogenesis contribute to excitotoxicity. Neurobiology of disease 43, 507–515, doi: 10.1016/j.nbd.2011.04.024 (2011). 21575722

[b26] Ghasemi M. & Schachter S. C. The NMDA receptor complex as a therapeutic target in epilepsy: a review. Epilepsy Behav. 22, 617–640 (2011). 22056342 10.1016/j.yebeh.2011.07.024

[b27] Hanada T. . Perampanel: a novel, orally active, noncompetitive AMPA-receptor antagonist that reduces seizure activity in rodent models of epilepsy. Epilepsia 52, 1331–1340, doi: 10.1111/j.1528-1167.2011.03109.x (2011). 21635236

[b28] Yen W., Williamson J., Bertram E. H. & Kapur J. A comparison of three NMDA receptor antagonists in the treatment of prolonged status epilepticus. Epilepsy research 59, 43–50, doi: 10.1016/j.eplepsyres.2004.03.004 (2004). 15135166 PMC2892717

[b29] Zahedifard M. . Synthesis, characterization and apoptotic activity of quinazolinone Schiff base derivatives toward MCF-7 cells via intrinsic and extrinsic apoptosis pathways. Sci Rep. 5, 11544 (2015). 26108872 10.1038/srep11544 PMC4479988

[b30] Cardoso A., Lukoyanova E. A., Madeira M. D. & Lukoyanov N. V. Seizure-induced structural and functional changes in the rat hippocampal formation: comparison between brief seizures and status epilepticus. Behav Brain Res. 225, 538–546 (2011). 21843555 10.1016/j.bbr.2011.07.057

[b31] Giardina W. J. & Gasior M. Acute seizure tests in epilepsy research: electroshock- and chemical-induced convulsions in the mouse. Curr Protoc Pharmacol. Chapter 5, Unit 5.22 (2009). 10.1002/0471141755.ph0522s45 22294398

[b32] Erakovic V., Zupan G., Varljen J., Laginja J. & Simonic A. Altered activities of rat brain metabolic enzymes in electroconvulsive shock-induced seizures. Epilepsia. 42, 181–189 (2001). 11240587 10.1046/j.1528-1157.2001.30499.x

[b33] Vezzani A., Ravizza T., Balosso S. & Aronica E. Glia as a source of cytokines: implications for neuronal excitability and survival. Epilepsia. 49 Suppl 2, 24–32 (2008). 18226169 10.1111/j.1528-1167.2008.01490.x

[b34] Willmore L. J. Antiepileptic drugs and neuroprotection: current status and future roles. Epilepsy & behavior: E&B 7 Suppl 3, S25–28, doi: 10.1016/j.yebeh.2005.08.006 (2005). 16239127

[b35] Rivera-Cervantes M., Feria-Velasco A. I., Junyent F., Espuny A. C. & Beas-Zárate C. Intracellular pathways associated with neuronal survival and death in epilepsy Pharmacoresistance in Epilepsy. pp. 77–97, Springer (2013).

[b36] Huberfeld G. . Glutamatergic pre-ictal discharges emerge at the transition to seizure in human epilepsy. Nat Neurosci. 14, 627–634 (2011). 21460834 10.1038/nn.2790

[b37] Johnson J. W. & Ascher P. Glycine potentiates the NMDA response in cultured mouse brain neurons. Nature. 325, 529–531 (1987). 2433595 10.1038/325529a0

[b38] Chapman A. G. Glutamate and epilepsy. J Nutr. 130, 1043s–1045s (2000). 10736378 10.1093/jn/130.4.1043S

[b39] Peterson S. L. & Albertson T. E. Neuropharmacology methods in epilepsy research: CRC Press (1998).

[b40] Henshall D. C. Apoptosis signalling pathways in seizure-induced neuronal death and epilepsy. Biochem Soc Trans. 35, 421–423 (2007). 17371290 10.1042/BST0350421

[b41] Lee C. H. . NMDA receptor structures reveal subunit arrangement and pore architecture. Nature. 511, 191–197 (2014). 25008524 10.1038/nature13548 PMC4263351

[b42] Miller P. S. & Aricescu A. R. Crystal structure of a human GABAA receptor. Nature 512, 270–275, doi: 10.1038/nature13293 (2014). 24909990 PMC4167603

[b43] Yelshanskaya M. V. . Structural Bases of Noncompetitive Inhibition of AMPA-Subtype Ionotropic Glutamate Receptors by Antiepileptic Drugs. Neuron 91, 1305–1315, doi: 10.1016/j.neuron.2016.08.012 (2016). 27618672 PMC5033713

[b44] de Vries S. J., van Dijk M. & Bonvin A. M. The HADDOCK web server for data-driven biomolecular docking. Nature protocols 5, 883–897, doi: 10.1038/nprot.2010.32 (2010). 20431534

[b45] Wassenaar T. A., Dijk M., Loureiro-Ferreira N. & Schot G. NMR: Structural Biology on the Grid. J. Grid. Comp. 10, 743–767 (2012).

[b46] Cardoso A., Carvalho L. S., Lukoyanova E. A. & Lukoyanov N. V. Effects of repeated electroconvulsive shock seizures and pilocarpine-induced status epilepticus on emotional behavior in the rat. Epilepsy & behavior: E&B 14, 293–299, doi: 10.1016/j.yebeh.2008.11.004 (2009). 19068237

[b47] Ferraro T. N. . Genetic influences on electrical seizure threshold. Brain research 813, 207–210 (1998). 9824700 10.1016/s0006-8993(98)01013-0

[b48] Ferraro T. N. . Mouse strain variation in maximal electroshock seizure threshold. Brain research 936, 82–86 (2002). 11988233 10.1016/s0006-8993(02)02565-9

[b49] Lloyd E., Somera-Molina K., Van Eldik L. J., Watterson D. M. & Wainwright M. S. Suppression of acute proinflammatory cytokine and chemokine upregulation by post-injury administration of a novel small molecule improves long-term neurologic outcome in a mouse model of traumatic brain injury. Journal of neuroinflammation 5, 28, doi: 10.1186/1742-2094-5-28 (2008). 18590543 PMC2483713

